# Overview of Membrane Science and Technology in Portugal

**DOI:** 10.3390/membranes12020197

**Published:** 2022-02-08

**Authors:** Liliana C. Tomé, Diogo M. F. Santos, Svetlozar Velizarov, Isabel M. Coelhoso, Adélio Mendes, João G. Crespo, Maria Norberta de Pinho

**Affiliations:** 1 LAQV-REQUIMTE, Department of Chemistry, NOVA School of Science and Technology, FCT NOVA, Universidade NOVA de Lisboa, 2829-516 Caparica, Portugal; liliana.tome@fct.unl.pt (L.C.T.); s.velizarov@fct.unl.pt (S.V.); jgc@fct.unl.pt (J.G.C.); 2 Center of Physics and Engineering of Advanced Materials (CeFEMA), Laboratory for Physics of Materials and Emerging Technologies (LaPMET), Chemical Engineering Department, Instituto Superior Técnico, Universidade de Lisboa, 1049-001 Lisbon, Portugal; diogosantos@tecnico.ulisboa.pt (D.M.F.S.); marianpinho@tecnico.ulisboa.pt (M.N.d.P.); 3 LEPABE—Laboratory for Process Engineering, Environment, Biotechnology and Energy, Faculty of Engineering, University of Porto, Rua Dr. Roberto Frias, 4200-465 Porto, Portugal; mendes@fe.up.pt

**Keywords:** membrane processes, membrane synthesis and characterisation, integrated (hybrid) solutions, sustainable approaches, circular economy, water–energy–material–food nexus, Portuguese membrane research

## Abstract

Membrane research in Portugal is aligned with global concerns and expectations for sustainable social development, thus progressively focusing on the use of natural resources and renewable energy. This review begins by addressing the pioneer work on membrane science and technology in Portugal by the research groups of *Instituto Superior Técnico*—*Universidade de Lisboa* (IST), *NOVA School of Science and Technology*—*Universidade Nova de Lisboa* (FCT NOVA) and *Faculdade de Engenharia*—*Universidade do Porto* (FEUP) aiming to provide an historical perspective on the topic. Then, an overview of the trends and challenges in membrane processes and materials, mostly in the last five years, involving Portuguese researchers, is presented as a contribution to a more sustainable water–energy–material–food nexus.

## 1. Introduction

Over the last few decades, considerable effort has been devoted to developing better membranes and extending their range of applications to different areas. Membrane processes already have an established role in gas separation and water treatment, and their applications in the food, pharmaceutical, and health areas have been continuously increasing.

In the last few years, membrane processes proved to have a key role in biorefinery and bioenergy production processes, namely for process intensification and the recovery and purification of valuable products. Membranes are also a crucial component of electrochemical energy conversion devices, including fuel cells and electrolysers.

Moreover, the growing environmental concerns have drawn attention to the use of fossil-based polymers and toxic solvents for membrane fabrication. Therefore, the development of new membranes, using polymers from renewable sources and more sustainable fabrication methods, is being pursued.

In Portugal, active membrane-related research is now being performed in a variety of domains, ranging from the production of high-quality drinking water to agriculture and irrigation use, the recovery of valuable compounds from natural sources and industrial effluents, and membrane reactors to energy generation and storage, as well as the integration of membranes and membrane technology in carbon capture and utilisation strategies.

This review begins by addressing the pioneer work on membrane science and technology in Portugal by the research groups of *Instituto Superior Técnico*—*Universidade de Lisboa* (IST), *NOVA School of Science and Technology*—*Universidade Nova de Lisboa* (FCT NOVA) and *Faculdade de Engenharia*—*Universidade do Porto* (FEUP) aiming to provide an historical perspective on the topic. Then, an overview of the trends and challenges in membrane processes and materials, mostly in the last five years, involving Portuguese researchers, is presented as a contribution to a more sustainable water–energy–material–food nexus. 

## 2. Historical Perspective on Membrane Science and Technology

### 2.1. Instituto Superior Técnico—Universidade de Lisboa (IST)

In early times, synthetic membranes were often associated with filtration and separation unit operations and, on a smaller scale, with a myriad of applications, such as battery separators, biosensors, controlled release systems, medical devices, etc. The huge developments in the 1960s and 1970s at the level of polymeric membranes and membrane modules allowed them to be implemented on an industrial scale of a vast number of membrane processes that required the mechanisms underlying the performance of this enormous diversity of membrane materials, structures and processes to be approached systematically. This was the driving force that moved Peter Bungay (National Institutes of Health), Harry Lonsdale (Founder of Journal of Membrane Science) and Maria Norberta de Pinho (IST) to organise (June 26–July 8) a NATO Advanced Study Institute (ASI) on “Synthetic Membranes: Science, Engineering and Applications” in Portugal in 1983. The book edited by the Institute organisers [1], in addition to numerous citations in Google Scholar, earned the following comment in Polymer News: “an outstanding contribution to the educational aspects of membrane science. In fact, if there are senior-level courses on membrane science, this is, the ‘textbook’ to use”. This NATO–ASI congregated and interconnected the major membrane groups of Europe and the U.S.A. and was the precursor of the “European Society for Membrane Science and Technology”. 

By that time, the Membrane group of the Chemical Engineering Department of Instituto Superior Técnico (IST), under the guidance of Maria Norberta de Pinho, progressed into two main lines of research: (1) Synthesis and Characterisation of Polymeric Membranes; (2) Design and Optimisation of Membrane Processes in Water, Food, Pulp and Paper, Cork and Chemical Industries.

From the first line of research, it is of particular interest to mention the synthesis of (1.1) Bi-Soft Segment Polyurethane Membranes for Pervaporation [2], which were further investigated (a project sponsored by the Portuguese Foundation for Science and Technology (FCT), 2003–2006) in terms of different soft segments towards the development of hemocompatible membranes for extracorporeal blood oxygenation [3], being the object of European (WO 2008/041126), U.S.A. (US2010/0111761) and Singapore (GST registration No: 200004489C) patents; (1.2) Integral Asymmetric Cellulose Acetate/Silica Hybrid Membranes for the selective removal of blood toxins in the Artificial Kidney [4]; and (1.3) Nanocomposite Membranes of Cellulose Acetate/Silver Nanoparticles and Cellulose Acetate/Silver Ion Exchanged Zeolites with bactericide activity for biofouling control [5].

In the second line of research, the design of membrane hybrid processes was carried out in the framework of European and national projects with industrial partners such as those coordinated by IST (scientific coordination of Maria Norberta de Pinho): “Treatment and Fractionation of Bleach Plant Effluents by Membrane Based Processes” (BRITE-EURAM(DGXII-EU)(1991–1994)) [6,7]; ”Integrated Process for Purification/Recovery of Ammonium Containing Water in Coke Plants” (EC(DG XVII)/ESSC Coal Research(1997–2000)) [8,9]; ”Valorization & Treatment of the Cork Industry Wastewaters” (JNICT/DGA(1996–1999)) [10]; ”Wine Must Concentration and Rectification by Nanofiltration and Electrodialysis“ (FCT/POCTI(2005–2008)) [11].

The assessment of tannic-ultrafiltration (UF) membranes’ interactions and membrane fouling characteristics have led to the development of integrated processes of flocculation/flotation/UF and ozonation/UF for tannins recovery from cork processing wastewaters [10,12,13]. Furthermore, nanofiltration (NF) concentrates were used as vegetal tanning agents in the leather industry [14]. UF and NF have also been investigated for the tertiary treatment of leather industry effluents [15,16,17]. In the pulp and paper industry, pioneering work was carried out on water recovery by the NF of bleach plant effluents and the development of hybrid processes of NF/ED and flotation/UF [6,7,18,19,20,21,22].

The prediction of the membrane processes performance through the modelling of the selective mass transfer was carried out in the membranes (surface forces/pore flow model) and at the membrane/fluid interfaces (quantification of concentration polarisation through mass transfer coefficients) for pressure-driven and pervaporation processes [23,24,25]. Flow management and mass transfer in spiral-wound modules with recourse to computational fluid dynamics (CFD) were given special attention by the team of Viriato Semião, Vitor Geraldes and Maria Norberta de Pinho [26]. The membrane characterisation by spectroscopic (attenuated total reflection-Fourier-transform infrared spectroscopy (ATR-FTIR), X-ray photoelectron spectroscopy (XPS)) and microscopic (scanning electron microscopy (SEM), transmission electron microscopy (TEM), atomic force microscopy (AFM)) techniques provided data that were correlated to the membrane selective permeation properties [27,28].

From 2004 to 2009, the membrane groups of IST and Nova School of Science and Technology—*Universidade NOVA de Lisboa* (FCT NOVA) were members of the European Network of Excellence (Sixth Framework Programme): “Expanding Membrane Macroscale Applications by Exploring Nanoscale Material Properties”, coordinated by Gilbert Rios at Institut Européen des Membranes. Divulgation activities were carried out, such as the “Membranes in Medicine“ workshop.

The present research at IST addresses: (1) The role of water dynamics in the membrane pores (investigated through deuterium and proton nuclear magnetic resonance (NMR)) on the selective permeation properties; (2) UF/NF membranes with bactericide activity and biofouling control in drinking water production; (3) Water recovery and sub-products valorisation in cork and food industries, namely polyphenols and polysaccharides in the wine industry; (4) Case-specific mass transfer correlations; (5) Minimisation of concentration polarisation and fouling prevention; (6) Microfluidics and membrane microdevices for the artificial kidney and artificial lung.

### 2.2. NOVA School of Science and Technology—Universidade Nova de Lisboa (FCT NOVA)

Membrane research at FCT NOVA started with the PhD work of João Crespo, which was focused on the study of different bioreactors for the simultaneous production of organic acids and vitamin B12 [29]. The first contact with membranes was related to the development of membrane bioreactors in a period (1980s) where this technology was in its initial stages. We look back with a smile when we remember that João complained to his supervisor that nobody was working, or interested, on that subject. Supervisors are sometimes right! The subject of membrane bioreactors has been a hot subject in the group over the years, namely through exploring other mechanisms of transport, as in the ion-exchange membrane (bio)reactor concept, which we proposed for the first time together with Maria Reis [30], and further developed with Svetlozar Velizarov [31,32,33], and the development of membrane biphasic enzymatic reactors in collaboration with Elias Klein and Carlos Afonso [34,35].

In the first years of the group (early 1990s), the collaboration with Karl Böddeker (GKSS, Geesthacht)—with whom we collaborated for many years working on organophilic pervaporation (Ph.D. project of Thomas Schäfer) [36,37]—and later with John Howell (University of Bath) [38] on the concept of critical/sustainable flux for the fractionation of proteins, was extremely inspiring and fueled our research for the coming years.

The membrane group at FCT NOVA grew significantly over the years, hopefully with a few relevant contributions for the community, namely on the understanding of transport mechanisms in membrane contactors (including a pioneer work on the use of ionic liquids in membrane contactors, which started with the PhD project of Isabel Coelhoso [39,40,41] and later with Raquel Fortunato [42,43] and Luísa Neves [44,45]) and, more recently, new approaches to membrane contactors for protein crystallisation [46] and production of nanoemulsions [47]. We have a particular interest in the development of techniques for monitoring membrane processes, using online and real-time approaches (namely by Mass Spectrometry, started by Thomas Schäfer [48] and continued by Carla Brazinha [49,50]), and using fluorescence techniques, namely through natural fluorescence (initial work with Gundula Wolf [51,52] and Carla Portugal [53,54], continued by Cláudia Galinha [55]) or using specific molecular probes. Soon, we concluded that machine learning approaches are essential for dealing with complex monitoring signals and large data sets (see the work of Gundula Wolf and Cláudia Galinha [56,57]). More recently, we became interested in the development of membranes with a designed surface topography (work of Pavel Izak [58] and later of Sylwin Pawlowski [59]), aiming to improve mass and heat transfer at the membrane surface (where it matters!), and the use of natural (bio)polymers for membrane manufacturing.

The membrane group of NOVA is now hosted in two different locations: at the research centre, Associated Laboratory for Green Chemistry (LAQV-REQUIMTE, the largest research centre in the country, which is focused on research and education in Sustainable Chemistry and Processes) at the faculty campus in Caparica; and at the private not-for-profit research centre iBET, in Oeiras, where the lab directly coordinated by Vanessa Pereira works on the integration of (photocatalytic)membranes (with a relevant contribution on membranes development by Rosa Huertas) with advanced oxidation processes, aiming the elimination of resilient organic micropollutants from water/wastewater streams [60,61].

The research group’s involvement in education programmes should be emphasised, namely the European Master programme EM3E, later EM3E-4SW, coordinated by Andre Ayral, and the Doctoral programme, Eudime, coordinated by Enrico Drioli.

### 2.3. Faculdade de Engenharia—Universidade do Porto (FEUP)

Since the end of the 1990s, Adélio Mendes’ group at the University of Porto has been developing research on several topics of membrane separation technology. Since then, this group contributions to the membrane-related field have been based on four pillars: development of materials for the fabrication of membranes, such as carbon molecular sieve membranes [62,63,64,65,66,67], palladium and ceramic membranes [68]; study of membrane processes, such as membrane reactors for propyne hydrogenation [69] and methanol steam reforming [70,71,72], direct methanol fuel cells (DMFCs) [73,74] and proton exchange membrane (PEM) fuel cells [75]; fundamental studies, namely the development of phenomenological models and simulators for membrane separation processes [76,77], membrane reactors [78,79,80], PEM fuel cells [81,82], redox flow batteries [83,84]; and technological projects, namely the development of membrane processes for recovering cutting oil and extraction of beer flavours for introducing in de-alcoholised beer for partially recovering the original flavour [85,86,87,88].

The first membrane-related article of the group at the University of Porto was published in 2002 [89] and concerned the removal of volatile organic compounds, such as acetone, ethyl acetate and ethanol from the air, using GKSS (Geesthacht, Germany) polyetherimide (PEI) membranes coated with polydimethylsiloxane (PDMS). Regarding the preparation and characterisation of carbon molecular sieve membranes, the first work was published in 2004 by Lagorsse et al. [62]. Brandão et al. [90] studied the mass transport in composite PDMS membranes loaded with palladium nanoparticles. Several studies on the use of hollow fibre membranes in membrane contactors with amino acid salt solutions were also published for CO_2_ removal in anaesthetic circuits [91,92,93]. In 2004, Janknecht et al. [85] studied the use of micro and ultrafiltration membranes for the removal of industrial cutting oil from oil emulsions.

The first Portuguese contribution to the field of direct methanol fuel cells membranes was reported by Silva et al. in 2005 [74]. Further work was developed in collaboration with Suzana Nunes from GKSS, who prepared sulfonated poly (ether ether ketone) (sPEEK) composite membranes with zirconium oxide for DMFC. Composite sPEEK membranes for DMFC with different additives were studied and compared with commercial Nafion^®^ membranes (DuPont, Wilmington, DE, USA) [94,95]. Brandão et al. [96] modified these Nafion^®^ membranes with Pd composite nanoclusters to reducemethanol crossover in DMFC. Concerning the development of PEMs for fuel cells, Boaventura et al. [75] prepared and characterised proton-conducting sulfonated polytriazole membranes. Studies on the utilisation, modification, activation and ageing of PEM fuel cells have also been developed [97,98,99,100,101,102].

Mendes’ research group’s future perspectives of CMS membranes separation technology are focused on developing a strategy to deliver this technology as quickly as possible for widespread use. A strategy to control the pore size for the different separations with industrial relief, such as the enrichment of oxygen or nitrogen from the atmospheric air, the separation of hydrogen from different streams, the separation of carbon dioxide from natural gas or biogas, among others, is currently being developed. Moreover, the use of supported hollow fibre CMS membranes is being investigated. In the future, pervaporation will also be a very relevant research topic in this technology.

Driven by the will of curbing down the notorious impact of burning fossil fuels on the global climate, currently, in Portugal, there are two spin-off companies from the University of Porto addressing membrane processes: SEA+TECH, an environmental, energy and engineering consulting company, which aims to develop energy-efficient and sustainable systems for the production, treatment, and recycling of water, for commercial or industrial applications; and CarboPora—a company targeting the development, production and commercialisation of membrane gas separation processes, namely oxygen-enriched air, CO_2_ removal from flue gas steams, methane separation/purification from biogas and hydrogen separation/purification from natural gas mixtures.

## 3. Activities on Membrane Science and Technology

This section describes the most recent activities on membrane processes and materials developed by Portuguese researchers in water treatment, food and health, energy and gas separation.

### 3.1. Water

Due to its extreme importance for the societies and ecosystems, and being at the core of their sustainable development, it is not surprising that membrane-assisted related studies for water treatment and reuse are a priority segment of the activities performed by Portuguese researchers. This is reflected by the number of studies published in the area of water treatment during the period 2017 to 2021 (13 articles related to drinking water and 41 articles related to various wastewater treatment applications by RO, NF and/or UF (Scopus)). Thus, a clear trend favouring wastewater-oriented applications is documented. This trend can be associated obviously with ever-increasingly stringent legislation criteria for wastewater discharge into the environment and with the quest for circular economy requiring water recycling and reuse. In this regard, NF (with 24 published articles) allowing a better trade-off between size-based and charge-based exclusion separation mechanisms, has attracted more attention.

#### 3.1.1. Drinking Water

Membrane processes (especially reverse osmosis (RO)) have proven to provide high-quality drinking water worldwide, using various water sources such as groundwater, surface water, and seawater, the latter especially in countries with limited in-land water bodies. In mainland Portugal, the water surface and groundwater sources are generally sufficient, and the drinking water-producing plants have well-developed infrastructure, allowing them to satisfy the required water demands. However, on the island of Porto Santo, an island of the Madeira Archipelago, the natural groundwater, especially during summer, become insufficient and therefore seawater desalination is applied. The plant includes four RO units with a total capacity of 6800 m^3^/d, operating at 50–65 bar [103]. In the framework of the operation of the Porto Santo plant, Geraldes et al. [104] carried out a techno-economic analysis of such medium-size RO plants for sea water desalination. Although it has a much smaller capacity of less than 1000 m^3^/d, RO-treated seawater has been used for irrigation and filling swimming pools in some hotels in Alvor (Algarve), thus saving the available drinking water resources for human consumption.

UF has proven to be an efficient pretreatment process for surface water before its use as a drinking water source. In Portugal, a SUEZ UF plant installed by the company Suez S.A. (formerly Suez *Environnement*) in Santa Eufémia (based north of Porto) treats water (with a capacity of 24,000 m^3^/d) from the nearby Ave River. It is operated by Vimágua, the municipal authority for water management in this region. This plant is one of the largest installations of Suez ZeeWeed 1500 modules in Europe, providing high-quality drinking water to roughly 80,000 people in the Guimarães and Vizela regions.

Since membrane processes provide several advantages, such as modularity, flexibility, ability to construct small size plants when compared to thermal desalination processes, both experimental and modelling studies have been performed by Portuguese researchers. Significant pioneering contributions include the preparation, by-phase inversion, and characterisation of dense and integrally skinned cellulose acetate (CA) and cellulose acetate butyrate membranes [28], and the development of an integrated model based on the finite volume formulation to numerically simulate the fluid flow in spiral-wound RO modules [26]. Afonso et al. [105] demonstrated that RO operated at 20–30 bar can highly reduce the content of organic and inorganic compounds (rejections >98.5%) present in raw waters brackish groundwater samples collected from the Zarqa basin, Jordan, at the cost of less than 0.30 EUR/m^3^.

Sanches et al. [106] studied the feasibility of NF removing pesticides and hormones from spiked distinct real water sources (surface water and groundwater) supplied by the drinking water company *Empresa Portuguesa das Águas Livres, S.A.* that treats and provides drinking water to approximately 2.8 million people in the region of Lisbon, and the effect of the water ionic composition on the rejection of target micropollutants. It was found that the NF efficiency in removing the selected compounds was not considerably affected by the conditions (static and or dynamic) of their pre-adsorption on the membrane.

The current trend in water-related membrane research is to develop integrated (hybrid) processes, which have already become preferred choices in several water treatment or usage applications. Exploring possible synergisms, benefiting from distinct process mechanisms, often leads to the development of novel, more efficient, and sustainable technologies to be applied in various domains, ranging from the production of clean water for human consumption to aquaculture and irrigation [107,108].

An integrated process (NF followed by direct or indirect UV photolysis) developed by Pereira et al. [60] has proven to be efficient for removing hormones with endocrine disruption capabilities from a real surface water matrix, thus guaranteeing the production of water of extremely high chemical quality.

A two-stage treatment to produce drinking water involving ion exchange and NF has been investigated [109]. In the first stage, the anion-exchange resin was used. A mathematical model that considers axial dispersion for the liquid phase predicted the breakthrough curves of Cl^−^ and SO_4_^2−^. The selectivity of separating Cl^−^, Na^+^, and SO_4_^2−^ ions by NF was assessed in the second stage.

**Figure 1:**
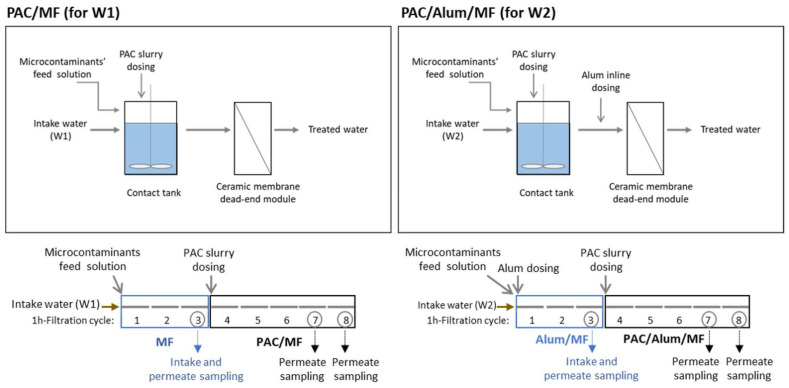
Scheme of powdered activated carbon/(coagulation)/microfiltration process, i.e., PAC/MF for intake water W1 and PAC/Alum/MF for intake water W2 (top) and of the respective spiking trials’ procedure (bottom) (8 × 1 h filtration cycles, with 3 cycles without PAC and 5 cycles with PAC dosing). Reprinted with permission from Campinas et al. [113]. Copyright 2021, MDPI.

Costa et al. [110,111,112] systematically investigated natural organic matter (NOM) removal and surface water treatment by UF and NF for the production of high-quality drinking water. More recently, the research group headed by Maria João Rosa at *Laboratório Nacional de Engenharia Civil* (LNEC) investigated powdered activated carbon (PAC)/coagulation/ceramic microfiltration (PAC/Alum/MF) treatment (Figure 1) on a pilot scale for treating low turbidity and low NOM surface waters spiked with various organic microcontaminants [113]. This treatment proved to be a reliable option for effectively removing turbidity (to below 0.03 NTU) while maintaining low residual levels of aluminium, which is an important advantage over the PAC conventional application.

A novel integrated drinking water treatment process referred to as ion-exchange membrane bioreactor (IEMB) has been introduced and comprehensively studied for the removal of various toxic inorganic anions (e.g., nitrate, perchlorate, bromate, arsenate, etc.) and heavy metals such as ionic mercury. The process concept is based on Donnan dialysis (DD) principles and consists of integrating Donnan dialytic transport of the target pollutant with its simultaneous precipitation, or selective biotransformation by suitable microbial culture to harmless metabolic products, in a separate receiver compartment [114]. The process operation was successfully validated with groundwater from the region of *Ponte de Sor*, Portugal, which is naturally polluted with arsenic. Its performance was recently optimised using the response surface methodology (RSM) approach, allowing the required operating conditions to be selected, which guarantee that the arsenic concentration in the treated water is below the maximum contamination level (MCL) allowed [115].

#### 3.1.2. Wastewater

##### Industrial Effluents

Regarded as a strategic pillar of the Portuguese economy, the agro-industrial sector (comprising primary agricultural production and the food and drinks industries) is extremely important and strongly contributes to the country’s rising productivity and competitiveness at the macroeconomic level [116]. In this regard, it is not surprising that the valorisation of the residues and by-products, and purification before discharge or re-use of the various aqueous effluents generated by this sector has gained considerable attention. Therefore, in this overview, they will be discussed in more detail. For wastewater originating from other industries, only two recent examples of studies performed by Portuguese researchers will be briefly presented.

The role of membrane processing in the agro-industry is discussed in detail in [117,118], and a few representative recent examples are briefly presented.

As an olive oil-producing country, Portugal generates a considerable amount of olive mill wastewater (OMW), which requires purification before discharge into natural water bodies and possible use for irrigation or re-use as process water.

Fraga et al. [119] reported extremely high degrees of total suspended solids and oil and grease removal when olive mill wastewaters were filtered using new silicon carbide tubular membranes. These membranes were used at constant permeate flux to treat real OMW at the pilot scale. Recently, the same researchers designed a new submerged membrane reactor with photocatalytic membranes prepared using the sol-gel technique that can be easily scaled up [120]. The proposed reactor is a promising approach to purify wastewaters of different origins. Combining inorganic membrane technologies with other novel methods such as advanced oxidation processes (e.g., using engineered nanomaterials) can be considered among the best options for dealing with highly polluted effluents [121].

In this regard, solvent cast and electrospun photocatalytic and antimicrobial membranes of poly(vinylidene fluoride-co-hexafluoropropylene) (PVDF-HFP) were prepared with different concentrations of TiO_2_ and Ag–TiO_2_ to produce a multifunctional material [122]. The recyclability of the membranes has also been demonstrated.

UF, either directly [123] or in combination with biological oxidation of the organics, present has been used for partial purification of OMW. In the latter case, a jet-loop aerobic bioreactor coupled with a UF membrane with a cut-off of 300 kDa is referred to as JACTO. MBR was designed, scaled up to 1000 L and tested in Portugal and Jordan [124]. To fulfil the legislation for irrigation in Jordan or for discharging to municipal wastewater treatment plants in Portugal, the treated OMW (UF permeate) in Portugal, an additional NF(RO) treatment should be applied to decrease the TOC values below the maximum allowed limits.

Using RO with a BW30 membrane, Antónia Nunes et al. obtained an acceptable TOC level in the RO permeate [125]. Still, its pH of 5.6 should be adjusted (increased) to comply with the acceptable range of 6.5–8.4 to be used as irrigation water.

Cork processing wastewater is another environmental problem due to its high organic matter content, such as sugars and non-biodegradable compounds such as polyphenols, namely tannins.

UF pre-treatment using cellulose acetate ultrafiltration membranes prepared by phase inversion was performed in concentration and diafiltration modes [126]. Still, to be used for irrigation, NF (RO) treatment of the UF permeates would be required.

NF and/or RO performances have been recently investigated for other types of agro-industrial wastewaters, such as NF for winery [127] and UF/RO for dairy wastewater [128]. In the latter study, the authors suggest using the RO permeates for cleaning purposes.

Portuguese researchers have also investigated the performance of membrane processes applied for purification and/or reuse of wastewater originating from other industries. Only two very recent examples of studies performed with real industrial effluents will be referred to in the following.

Having to constantly rely on access to great amounts of water, the oil and gas industry has sought opportunities to reclaim water using sustainable and efficient management procedures. An on-site pilot plant treatment study on real refinery stripped sour water using RO was conducted to remove the primary pollutant blocking the possibility for water reuse in the refinery cooling tower, phenols [129]. A technical and energy demand analysis was performed, in which 90% of the initial wastewater was recovered without loss of permeate quality. It was found that installing an energy recovery device with the RO plant can decrease the operating costs from EUR 1.37 to EUR 0.41 per m^3^ of treated water.

A pre-filtered real tannery effluent (collected from a plant in Algeria) was subjected to NF with (NF270 or NF90 membranes) membranes or RO (BW30 and SW30) membranes to compare their performance [130]. It was found that the RO permeate obtained with the SW30 membrane conforms to local Algerian regulations regarding being discharged directly into the natural environment (in this case, Reghaia Lake) or into urban sewers linked to wastewater biological treatment stations.

##### Sewage (Domestic Wastewater)

These water matrices contain liquid effluents from households and businesses, as well as pretreated or not industrial wastewater effluents, which makes their direct membrane-assisted purification extremely challenging. Therefore, membrane processes are generally combined with other treatment processes such as adsorption, precipitation, coagulation, oxidation, etc. Portuguese researchers have been actively developing and designing such integrated treatment technologies.

At LNEC, Viegas et al. [131] investigated the long-term performance of hybrid adsorption with Powdered Activated Carbon (PAC)—coagulation (with FeCl_3_)–ceramic membrane filtration (HCMF) process operated with a sand-filtered secondary effluent at pilot scale in two test sites, namely Frielas and Beirolas wastewater treatment plants, which are two of the biggest ones in Lisbon metropolitan area, with average daily flowrates of 54,500 m^3^/day and 70,000 m^3^/day, respectively. A detailed cost analysis of the process, as a function of the plant flow rate, was performed pointing to total production costs of 0.21 EUR/m^3^ for a 50,000 m^3^/day plant and 20 years’ membrane lifetime, with a higher share of OPEX (0.17 EUR/m^3^), mainly associated with equipment/membranes replacement and reagents costs than with CAPEX (0.04 EUR/m^3^). The membrane lifespan was shown to be a key factor in the costs.

An integrated hybrid system coupling microfiltration (MF) with UVA photolysis (UVA-MF) or TiO_2_-P25 photocatalysis (UVA/TiO_2_-MF) was investigated for the removal of oxytetracycline (OTC) from a real secondary effluent taken from a municipal wastewater treatment plant [132]. A significant reduction in the permeate flux and OTC removal degree was documented due to membrane fouling. Therefore, the authors pointed out that further research should focus on the possible addition of oxidants (H_2_O_2_, persulfate, or ozone) to improve the process performance.

**Figure 2:**
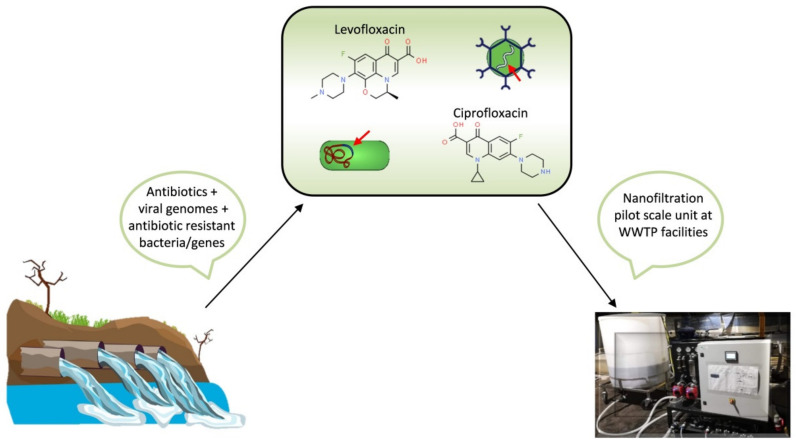
On-site removal of antibiotics, antibiotic resistance genes and viral genomes from a domestic wastewater effluent by a nanofiltration pilot unit. Reprinted with permission from Cristóvão et al. [133]. Copyright, MDPI.

NF was applied to pre-treated (filtered first with 1.2 µm filters and then with 0.45 µm polyamide membrane) domestic wastewater collected after the biological treatment and prior to the disinfection step and spiked with target antibiotics and viral genomes (Figure 2) [133]. Since high rejections were obtained, the authors consider NF a promising tertiary treatment option. In another study, Cristovão et al. [134] demonstrated the effectiveness of NF to remove several widely used anticancer drugs from hospital and wastewater effluents.

A combination of advanced oxidation technologies and direct contact membrane distillation (DCMD) was investigated to treat municipal wastewater collected from the secondary clarifier of a municipal wastewater treatment plant located in Northern Portugal, which receives both domestic and industrial effluents [135]. It was found that the dissolved organic carbon content in the wastewater strongly affects the permeate fluxes achieved by DCMD. In this regard, the authors concluded that a combination of ozonation and DCMD is more appropriate for wastewaters with low dissolved organic carbon DOC contents (<15 mg/L).

### 3.2. Membrane Applications in Food and Health

#### 3.2.1. Membrane Processes in Biorefinery

The biorefinery approach has enhanced the valorisation of agro-wastes reducing the environmental impact and creating added value products using sustainable practices in line with the principles of the circular economy. Membrane processes play a key role in bioactive compounds fractionation. They are an excellent alternative to conventional methodologies due to their low energy and no-additives requirements, mild conditions of temperature and pressure and easy scale-up. Pressure-driven membrane processes (microfiltration (MF), ultrafiltration (UF), nanofiltration (NF), and reverse osmosis (RO)) were largely explored by Portuguese researchers for the separation, purification, and concentration of bioactive compounds from several agri-food by-products, including grape pomaces [136,137], corn fibre and Eucalyptus bark [138,139].

Among wastes, grape pomaces are a valuable source of polyphenolic compounds (flavonoids, tannins, and benzoic acids derivatives), which can be further used for innovative formulations in the pharmaceutical and food industries. Arboleda et al. [136] evaluated the feasibility of phenolic compounds recovery from red grape pomace through a combination of ultrasound-assisted enzymatic extraction, followed by a membrane separation process. Different cellulose acetate membranes were prepared and tested. The CA400-22 membrane presented high retention coefficients towards phenolic compounds and proanthocyanidins, 73% and 92%, respectively, and exhibited low retention values for glucose and fructose (rejection values of 19.5% and 12.5%, respectively). The results indicate that combining an ultrasound-assisted enzymatic extraction with tailor-made cellulose acetate membranes can be a sustainable approach for recovering valuable fractions from red grape pomace.

Syed et al. [137] proposed an effective production of a grape pomace extract enriched in bioactive monomeric flavan-3-ols by an extraction process using 40 wt.% ethanol/water followed by dia-nanofiltration processing with a Duramem 900 membrane. The optimised fractionated extract was further concentrated by RO, allowing for the re-use of the hydro-ethanolic washing solution.

Giacobbo et al. [140,141] studied the recovery of wine polysaccharides and polyphenols through the fractionation of wine lees by pressure-driven membrane processes.

Corn fibre, a co-product of the starch industry, is rich in compounds with high added value, such as ferulic acid and arabinoxylans, released during alkaline extraction. Ferulic acid presents several benefits, namely antioxidant, anti-inflammatory, antiatherogenic and antiproliferative properties, making this compound widely used in the food, cosmetics, and pharmaceutical industries. Moreover, it can be used as a precursor of bio-vanillin production. Ultrafiltration was tested for separating ferulic acid from arabinoxylans due to the high molecular mass difference between the two compounds. In the work of Valério et al. [138], a Nadir UP 150 membrane was found to be an adequate choice, allowing for efficient separation of ferulic acid from arabinoxylans, with a null rejection of ferulic acid and high rejection of arabinoxylans 98%.

Polyphenols, extracted from lignocellulosic side-streams from the pulp and paper industry, have not received much attention concerning their fractionation and purification. Among the lignocellulosic materials, the bark is one of the most promising for high-added-value chemicals production due to its chemical composition and availability. Restolho et al. [142] studied the recovery of sugars and lignosulphonates from eucalyptus spent sulphite liquor by pressure-driven membrane processes. Pinto et al. [139] investigated the processing of ethanol: water extract of *Eucalyptus globulus* bark containing bioactive polyphenols by dia-ultrafiltration (DF) and adsorption/desorption process. Pre-concentration followed by DF with a 30 kDa membrane provided an increment of 21% and 64% of total phenolic compounds (TPC) and proanthocyanidins (Pac), respectively, relative to the initial extract composition. The resulting retentate was further purified by fixed-bed adsorption/desorption using a polymeric adsorbent (SP700). In the desorption step with ethanol: water 95:5% *v*/*v* about 45% of the carbohydrates were eliminated and an enriched fraction in polyphenols was obtained. Moreover, the integrated process also led to the recovery of low molecular weight phenolic compounds at the permeate stream during membrane processing and the recovery of galacturonic acid-rich carbohydrates coming from the washing step of the fixed-bed process.

#### 3.2.2. Valorisation of Food Industry Wastewaters and Microalgae

The fishing industry is a pillar of the economy for many countries worldwide. Fish processing by-products and underused catch containing a large quantity of protein are generally converted into low-value products such as animal feed and fertilisers. However, they can be a source of value-added compounds such as peptides and amino acids, capable of modulating physiological processes and, therefore, may have a role in the prevention and control of diseases. The work of Ghalamara et al. [143] demonstrated the feasibility of using (pressure-driven) membrane technologies to purify small biopeptides from codfish blood and sardine cooking wastewaters, the most processed species in Portugal. The best-performing membrane was a UP010, with a minimum rejection of 2% of the small peptides. Results of antioxidant activity suggest that codfish blood is a potential source of natural antioxidant peptides, which may be used as a functional food additive to reduce oxidative stress-associated diseases and inhibit oxidation in foods. Furthermore, the purified peptides also exhibited an interesting antimicrobial activity against *Escherichia coli*.

*Dunaliella salina* is well-known for its high content in carotenoids and glycerol. Nevertheless, *D. salina* also has a high content in polar lipids, suitable for nutraceutical applications. Monte et al. [144] proposed a sustainable process to produce distinct fractions of carotenoids, glycerol, polar lipids, and proteins, which may improve the microalgae industry’s revenues. This work integrates extraction with non-hazardous solvents and organic solvent nanofiltration to obtain added-value products and glycerol. High overall recoveries were obtained for carotenoids (85%), glycerol (86%), polar lipids (94%), and proteins (95%).

#### 3.2.3. Applications of Membranes in Health

Membrane processes have been used for protein crystallisation [46,145,146,147], and membrane contactors have been used for CO_2_ removal from anaesthesia gases [148,149,150] and as blood oxygenators [151,152,153].

**Figure 3:**
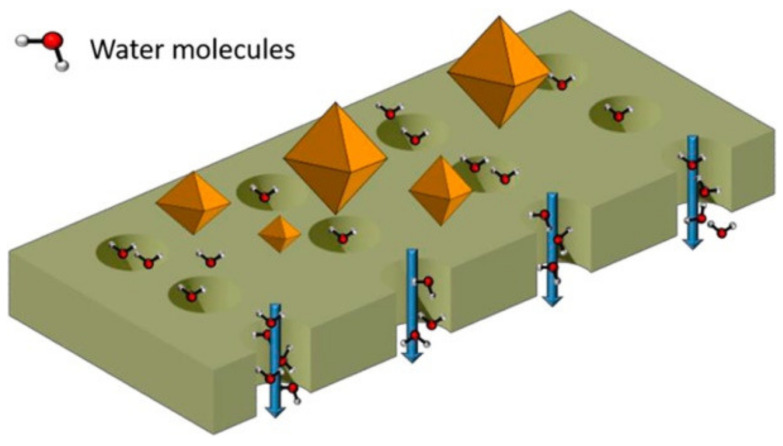
Membrane-assisted crystallisation principle. Protein and stripping solution are on different sides of the membrane. The volatile solvent is removed from the protein solution in the vapour phase; supersaturation is reached, and crystals form. Reprinted with permission from Polino et al. [145]. Copyright 2019, American Chemical Society.

Crystallisation kinetics can be enhanced by playing with membranes’ morphology, topography, and chemistry, allowing for tailoring the supersaturation rate and inducing nucleation at a lower degree of supersaturation. Furthermore, a controlled diffusion of compounds during post-crystallisation treatments leads to higher crystal stability avoiding crystal handling. The use of small volumes, laminar flow, and low-cost fabrication processes of microdevices are extremely important; however, few studies are currently available regarding the use of microtechnology for protein crystallisation. Polino et al. [146] used a Nafion^®^ membrane (Sigma-Aldrich, 1100EW, St. Louis, MI, USA) integrated with a polydimethylsiloxane (PDMS) microdevice for protein crystallisation of Hen Egg White Lysozyme (HEWL) purchased from Sigma-Aldrich and subsequent in situ crystal derivatisation based on efficient control of the diffusion rate of the derivatising agent (Hg^2+^), assured by the ion-exchange membrane (Nafion^®^117) (Sigma-Aldrich, 1100EW, St. Louis, MI, USA) integrated into the microdevice. These conditions were key to guaranteeing crystal structural integrity along the derivatisation process, proving to be a more efficient alternative to the traditional crystal soaking methodologies (Figure 3).

The noble gas xenon possesses many characteristics of an ideal anaesthetic agent. However, its application is limited in daily clinical routine due to its elevated price. Martins et al. [148] propose using a gas-ionic liquid membrane contactor system, using a cholinium lysinate ionic liquid solution as CO_2_ absorbent, for the removal of carbon dioxide in anaesthesia closed-circuits, allowing the recycling of the anaesthetic agents. The ability of the proposed system to recover xenon drastically reduces the cost, which can be a step forward for the routine use of this gas in surgeries.

Due to their high hemocompatibility and gas permeation capacity, bi-soft segment polyurethane/polycaprolactone (PU/PCL) polymers are promising materials for use in membrane blood oxygenators. Eusébio et al. [151] synthesised both nonporous symmetric and integral asymmetric PU/PCL membranes. The permeation properties of the atmospheric gases N_2_, O_2_, and CO_2_ through these membranes were experimentally determined using a new custom-built gas permeation apparatus.

Oxygen mass transfer in a membrane blood oxygenator (MBO) surrogate system has been addressed by Faria et al. [152]. It consists of a slit for water circulation as a surrogate blood flow channel and a constant pressure oxygen chamber separated by an integral asymmetric hemocompatible polyurethane-based membrane. The experimental fluxes are in very good agreement with the predictions based on unidimensional axial convection and unidimensional transversal diffusion. This custom-made benchmark system will allow the flow and oxygen mass transfer to be optimised, in order to design a novel flat-sheet MBO.

**Figure 4:**
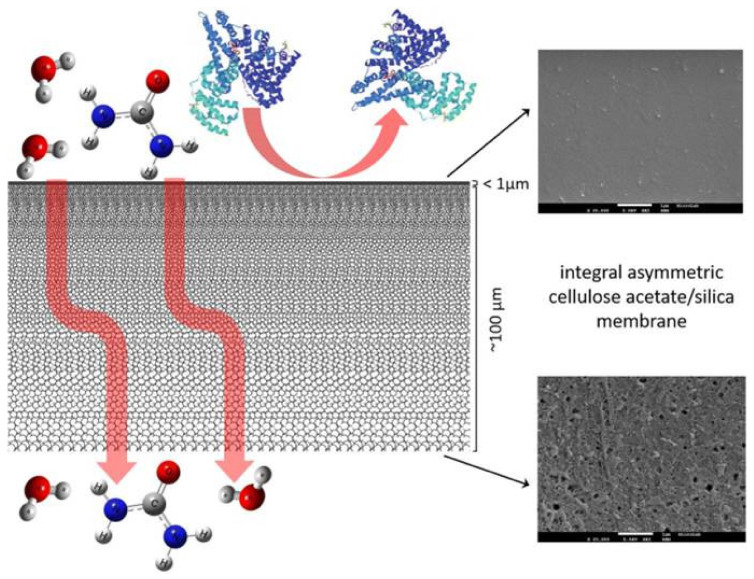
Monophasic hybrid cellulose acetate/silica (CASiO_2_) integrally skinned membranes with tailored hemocompatible surfaces. Reprinted with permission from Faria et al. [153] Copyright 2020, Springer Nature.

Faria et al. [153] synthesised by an innovative method, combining phase inversion with the acid-catalysed sol-gel process, monophasic hybrid cellulose acetate (CA) membranes with tailored hemocompatible surfaces and permeation properties that assure the kidney metabolic functions of preferential permeation of uremic toxins and the retention of vital components of the blood such as albumin (Figure 4). Permeation studies show that the integration of silica into CA membranes results in high flux UF membranes with enhanced mass transfer properties on the metabolic functions of the kidney. The hemolysis assay shows that all the hybrid CASiO_2_ membranes are non-hemolytic and that, even for an extended static blood/membrane surface contact time of 15 min, the thrombosis degree exhibited by all the membranes is lower than 30% of the value observed for the positive control. Shear stresses induced by UF permeation have also been analysed in a surrogate system of an artificial kidney [154].

### 3.3. Membranes for Energy Applications

#### 3.3.1. Membranes for Fuel Cells

The membrane is a crucial component for most electrochemical energy conversion and storage devices, including batteries, electrolysers, and fuel cells. The latter are truly sustainable and clean energy technologies, where hydrogen and oxygen combine to generate electricity, water, and heat. Proton-exchange membranes (PEMs) remain a critical material and key challenge to fuel cell science and technology development. Further advances are urgently required, as PEM fuel cells demand increases with the fuel cell electric vehicles market growth.

In this regard, Teixeira et al. [155] doped Nafion membranes to enhance proton conductivity in PEM fuel cells. The dopants were derived from aryl mono- or bisphosphonic acid and incorporated using impregnation or casting methods. Electrochemical impedance spectroscopy (EIS) was used to assess the proton conductivity in these membranes and evaluate the influence of structure and preparation method on proton transport. The membranes prepared by casting showed higher proton conductivities than commercial Nafion^®^N-115 (FuelCell Store, College Station, TX, USA) at all temperatures and relative humidity conditions, with [1,4-phenylenebis(hydroxymethanetriyl)]tetrakis(phosphonic acid) offering the best proton conductivity (87 mS cm^−1^) [155]. In another study, Teixeira et al. [156] prepared different indazole- and benzotriazolebisphosphonic acids for incorporation into Nafion^®^N-115 membranes (FuelCell Store, College Station, TX, USA) (up to 5 wt.% loading). Nafion-azolebisphosphonate membranes with 1 wt.% loading presented enhanced proton conductivity compared to benchmark Nafion^®^N-115, with the membrane doped with [hydroxy(1H-indazol-3-yl)methanediyl]bis(phosphonic acid) exhibiting a proton conductivity of 98 mS cm^−1^. In both studies, the results suggest that Grotthuss and vehicular mechanisms are involved in the proton conduction across the membrane. The good proton conductivity of these doped membranes led Teixeira et al. [157] to evaluate their potential application in PEM fuel cells. Membrane-electrode assemblies (MEAs) containing the membrane doped with [1,4-phenylenebis(hydroxymethanetriyl)]tetrakis(phosphonic acid) presented better performance than Nafion^®^N-115 at all studied temperatures, with a maximum power density of ~383 mW cm^−2^ at 70 °C.

Natural polymers, such as cellulose, the most abundant biopolymer, and in particular its nanoscale forms (e.g., cellulose nanocrystals (CNCs), cellulose nanofibrils (CNFs), bacterial nanocellulose (BNC)) have been proposed as appropriate materials to engineer the membrane of PEM fuel cells [158]. Vilela et al. [159] focused on the potential of BNC as a substrate for the design of bio-based ion-exchange membranes, as they present an excellent combination of conductive and mechanical properties. In this context, they fabricated polyelectrolyte nanocomposite membranes based on poly(bis [2-(methacryloyloxy)ethyl] phosphate) [P(bisMEP)] and BNC via the in situ free radical polymerisation of bis[2-(methacryloyloxy)ethyl] phosphate (bisMEP) inside the BNC three-dimensional network. Vilela et al. also produced other BNC-based nanocomposites with poly(methacryloyloxyethyl phosphate) [160] and even fully bio-based ion-exchange membranes by blending BNC either with lignosulfonates [161] or algae sulphated polysaccharide (i.e., fucoidan) [162], together with tannic acid as a natural crosslinker.

In addition, Vilela et al. [163] prepared polyelectrolyte nanocomposites of BNC and poly(4-styrene sulfonic acid) as eco-friendly PEMs for microbial fuel cells (MFCs). The application of the nanocomposite membrane in a single-chamber lab-scale MFC with a pure culture of *Shewanella frigidimarina* yielded a maximum power density of 2.42 mW m^−2^, open-circuit voltage of 0.436 V, and internal resistance of 15.1 kΩ, which was better than that obtained with a commercial Nafion^®^117 membrane. In another work, Vilela et al. [164] developed bio-based anion-exchange membranes (AEMs), suitable for application in alkaline fuel cells. The nanocomposite membranes were based on a tetraalkylammonium-derived polymeric ionic liquid, i.e., poly(methacroylcholine chloride), and BNC.

Recently, Ferreira et al. [165] used computational fluid dynamics (CFD) to simulate the membrane chemical degradation in PEM fuel cells. They found that degradation accelerated when voltage, temperature and pressure increased and when reactants’ humidity and membrane thickness decreased.

Direct liquid fuel cells (DLFCs) are receiving increasing attention due to their advantages over conventional hydrogen fuel cells. These include higher energy density, easier transportation and storage, simplified structure, and the possibility of using a small fuel cartridge for instant recharging. Alcohols (e.g., methanol, ethanol) and sodium borohydride are the most common organic and inorganic fuels used in DLFCs.

Electrolyte membranes that restrict the methanol crossover while retaining proton conductivity are essential to improving the electrochemical selectivity in direct methanol fuel cells (DMFCs). Rambabu et al. [166] reviewed the literature on carbon nanomaterial-based PEMs published during the last decade. Based on the reported proton conductivity and methanol permeability of nanocomposite membranes containing carbon nanotubes, graphene oxide and fullerene as additives, Rambabu et al. [166] critically assessed the impact of each type of filler on those properties.

Ben Jadi et al. [167] prepared Nafion-polypyrrole membranes by a straightforward deposition method and assessed their transport properties for DMFC application. The effect of the polypyrrole coating on transport properties (e.g., water uptake, ion-exchange capacity, proton conductivity) was investigated. They found that methanol diffusion through the membrane was reduced by at least 20-fold compared to commercial Nafion^®^112. Ben Jadi et al. [168] also synthesised Nafion^®^112/polyaniline composite membranes for use in DMFCs, which led to a decrease in the methanol crossover and an increase in the proton conductivity compared to the unmodified Nafion^®^112.

Oliveira and Mendes [169] prepared eco-friendly polymer electrolyte membranes based on a blend of sulphonated poly(vinyl alcohol)/chitosan stabilised by nylon 6,6, using sulfosuccinic acid both as a crosslinker and sulfonating agent. The membranes presented good proton conductivity (ca. 10^−2^ S cm^−1^) and lower H_2_ and methanol permeability than benchmark Nafion^®^115.

Direct borohydride fuel cells (DBFCs) are a subtype of DLFCs and an excellent alternative to alcohol-fed fuel cells, as their operation does not emit greenhouse gases. DBFCs operate with sodium borohydride as the fuel and oxygen or hydrogen peroxide as oxidants. They offer advantages over other DLFCs regarding higher energy density and theoretical cell voltage [170]. To reach the initial stages of DBFCs commercialisation, it is still necessary to develop more efficient and lower-cost membranes.

Gouda et al. [171] produced composite membranes via polymer crosslinking and casting techniques. A ternary crosslinked polymer was prepared from low-cost and readily available polymers containing poly(vinyl alcohol) (PVA), poly(ethylene oxide) and poly(vinyl pyrrolidone), followed by incorporation of sulphonated graphene oxide (SGO) as a doping agent into the polymer matrix. The prepared membranes presented half the borohydride permeability of Nafion^®^117. In another work, Gouda et al. [172] doped the same PVA-based ternary blend with (PO_4_–TiO_2_) and (SO_4_–TiO_2_) nanotubes. The DBFCs operating with both types of membranes attained power densities close to that recorded with a Nafion^®^117 membrane, but at a significantly lower membrane cost [171,172]. Those results pave the way for a green, low-cost and straightforward approach to developing composite membranes for practical DBFCs.

#### 3.3.2. Membranes for Batteries

Batteries are everywhere, from flashlights to remote controls, from mobile phones to laptops. Specifically, lithium-ion batteries (LIBs) are seeing an increasing demand due to the boom of battery electric vehicles. This requires the development of advanced materials to improve LIBs’ energy/power density, conversion efficiency and safety, as well as decrease costs, extend their lifetime, and reduce their environmental impact.

The membrane separator plays a key role in LIB performance, separating the electrodes and controlling the movement of the lithium ions. Due to their suitable properties, polymer composites and blends have been frequently used as LIB separators. Nunes-Pereira et al. [173] prepared porous poly(vinylidene fluoride-co-trifluoroethylene) (PVDF-TrFE) based composite membranes filled with clays (montmorillonite), zeolites (Y zeolite), ceramics (barium titanate) and carbonaceous (multiwalled-carbon nanotubes) fillers. The results demonstrated that the filler type deeply affected the membrane separator performance in LIBs. The PVDF-TrFE membrane with MMT filler presented the best performance among all the evaluated fillers.

Costa et al. [174] prepared silica/PVDF porous composite membranes for LIB separators using a non-solvent induced phase separation process with different air exposure times before immersion in a water coagulation bath and filler content of 20 wt.%. Mesoporous silica spheres (SS) of ~400 nm average diameter were synthesised using the sol-gel method and dispersed into the polymer matrix. It was demonstrated that the morphology, degree of porosity, uptake value and electrical properties of the composite membranes were influenced by the exposure time to air and the presence of SS. The membranes were assembled in Li/C-LiFePO_4_ half-cells, and the best cycling performance was obtained for the composite membrane after 1 min exposure to air, with an ionic conductivity of 0.9 mS cm^−1^.

Cellulose has emerged as a sustainable alternative to traditional polyolefin-based LIB separators because of its good mechanical properties and inherent hydrophilic character. Gonçalves et al. [175] fabricated high specific surface area mesoporous cellulose nanocrystal membranes with different pore morphology as novel three-dimensional porous separators. These membranes displayed ionic conductivities of 2.7 mS cm^−1^, wide electrochemical stability, and good interfacial compatibility with the lithium electrode, delivering a specific capacity of 122 mAhg^−1^ and 85 mAhg^−1^ at a C/2 and 2C rate, respectively.

Barbosa et al. [176] produced electrospun PVDF fibre membranes doped with different ionic liquids sharing the same anion, bis(trifluoromethylsulfonyl)imide [TFSI]. Their potential as membrane separators for LIBs was evaluated. The membrane doped with 15% 1-ethyl-3-methylimidazolium([C_2_mim])[TFSI]) showed the best performance, with an ionic conductivity of 2.8 mS cm^−1^ at room temperature and good stability in the electrochemical window 0.0–6.0 V. When evaluated in C-LiFePO_4_ half-cells, it exhibited a maximum discharge capacity of 119 mAhg^−1^ at C-rate, with a coulombic efficiency close to 100%.

Rechargeable zinc (Zn)-air batteries are seen as attractive candidates for large-scale electricity storage owing to their high volumetric energy density, safety, economic feasibility, and environmental friendliness. In Zn-air batteries, the membrane allows the transport of OH^−^ ions between the air electrode and the Zn electrode while providing a physical barrier that prevents short-circuiting. Zn-air battery performance is greatly affected by the physicochemical properties of the employed membrane. However, the development of appropriate membranes has received insufficient attention. Tsehaye et al. [177] critically discussed the state-of-the-art membranes for Zn-air batteries. They presented membranes synthesis and modification strategies to minimise the crossover of zincate ions and the formation/growth of Zn dendrites. The work provides guidance for researchers and industries for selecting adequate membranes to commercialise rechargeable Zn-air batteries.

The proliferation of portable electronic devices has increased the amount of e-waste generated after their use. One of the most hazardous components in e-waste is batteries due to the heavy metals and toxic chemicals that they contain. Fuel cells and redox flow batteries are sustainable alternatives to LIBs for powering portable applications. Although they provide comparable energy and power density, they face challenges related to their dependence on PEMs based on non-environmentally friendly, high-priced perfluorosulfonic acid copolymers, which require energy-intense manufacturing and recycling procedures. Alday et al. [178] synthesised eco-friendly and sustainable biopolymer electrolyte membranes (BioPEMs) from biopolymers such as chitosan, cellulose, and starch. These BioPEMs present advantages in performance, sustainability, and cost. They also show good chemical and mechanical stability, and ionic conductivities close to that of Nafion membranes. Redox flow batteries using cellulose–chitosan-based BioPEMs delivered open-circuit voltages above 0.75 V and power density up to 2.5 mW cm^−2^. These BioPEMs show great promise in improving biodegradable batteries in sectors requiring transient electrical energy, such as environmental monitoring, agriculture, or packaging.

Minimising the redox species crossover through the membrane is a key challenge, as it leads to capacity loss and lower coulombic efficiency. This issue needs to be readily quantified before commercial application of the non-aqueous redox flow battery (NAqRFB). Thus, Mushtaq et al. [179] developed in situ crossover diagnostics to assess the membrane efficacy in NAqRFB. The successful validation of this technique enables its use as a reference testing method to measure crossover and help select the best performing membrane for a redox flow battery. As a membrane diagnosis tool, this technique has the advantage of in situ determination of crossover without the need for disassembling the devices.

#### 3.3.3. Reverse Electrodialysis for “Blue Energy” Generation

Reverse electrodialysis (RED) is a membrane-based technology that allows salinity gradient energy to be harvested. Fouling is an undesirable operation constraint in the RED process since it decreases obtainable net power density due to increasing stack electric resistance and pressure drop. Therefore, early fouling detection is one of the main challenges for successful RED technology implementation. S. Pawlowski et al. [180] used two-dimensional (2D) fluorescence spectroscopy as a tool for fouling monitoring in RED. Fluorescence excitation-emission matrices (EEMs) of ion-exchange membrane surfaces and natural aqueous streams were acquired during one month of a RED stack operation. The fouling evolvement on the ion-exchange membrane surfaces was followed by 2D fluorescence spectroscopy and quantified using principal components analysis (PCA). The authors found that the AEM surface in contact with river water was significantly affected due to fouling by humic compounds crossing the membrane from the lower salinity (river water) to the higher salinity (seawater) stream. The combination of 2D fluorescence spectroscopy and PCA demonstrated a high potential for studying fouling development and membrane cleaning efficiency in ion-exchange membrane processes.

Still, a major hindrance to the implementation of RED is the high price of ion-exchange membranes. S. Pawlowski et al. [59] proposed using profiled membranes to overcome the shadow effect of non-conductive spacers, which reduce the membrane area available for counter-ion transport and increase the stack electric resistance. Profiled membranes can be a promising alternative as the reliefs formed on their surface keep the membranes separated and provide channels for the solutions to flow. S. Pawlowski et al. [59] also used CFD to simulate fluid behaviour in channels formed by various profiled membranes. The highest net power density values were obtained for the corrugation shape and arrangement in the form of chevrons due to the increase in the available membrane area and an excellent balance between enhancement of mass transfer and the increase in the pressure drop in the channel. When properly designed, corrugated membranes offer better performance than conductive spacers. The proposed membrane corrugation design can be extended to other electromembrane processes, such as electrodialysis and Donnan dialysis, where high ionic mass transport rates are desirable at as low as possible energy costs [59].

**Figure 5:**
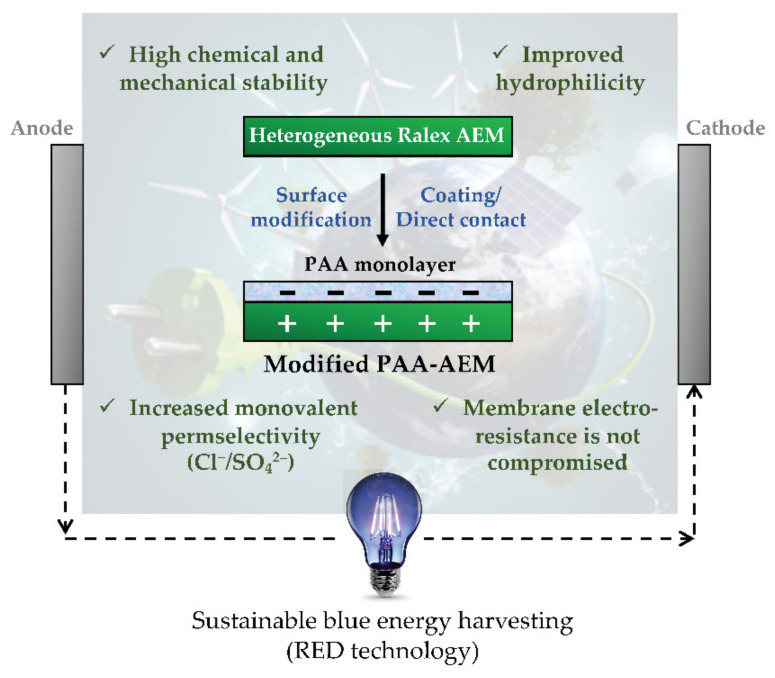
Scheme of the procedure used to functionalise Ralex-AEMs with poly(acrylic) acid and the advantages of the PAA modified AEMs for sustainable blue energy harvesting [181].

The performance of AEMs in RED can be hampered by the presence of multivalent ions and fouling phenomena, thus leading to reduced net power density. Merino-Garcia et al. [181] proposed a monolayer surface modification procedure to functionalise Ralex-AEMs with poly(acrylic) acid (PAA) to (i) render a monovalent permselectivity, and (ii) minimise organic fouling. Membrane surface modification was carried out by putting heterogeneous AEMs in contact with a PAA-based aqueous solution for 24 h (Figure 5). The membranes’ permselectivity and fouling behaviour in the presence of humic acid was evaluated through mass transport experiments using model NaCl-containing solutions. The use of PAA-modified AEMs resulted in a significantly enhanced monovalent permselectivity (sulphate rejection improved by >35%) and membrane hydrophilicity (contact angle decreased by >15%) in comparison to the unmodified Ralex-AEMs, without compromising the membrane electro-resistance after modification. The work demonstrates the proposed membrane modification procedure’s feasibility and provides a path for improving RED efficiency.

#### 3.3.4. Membranes for Electrolytic Hydrogen Production

Water splitting has been proposed as a promising approach to producing clean hydrogen. The two half-reactions of water splitting, the hydrogen evolution reaction (HER) and oxygen evolution reaction (OER), occur kinetically fast in solutions with completely different pH values. Enabling HER and OER to occur under kinetically favourable conditions while using exclusively low-cost, earth-abundant electrocatalysts is highly desirable, but remains a challenge.

J. Xu et al. [182] demonstrated that using a bipolar membrane (BPM), it is possible to carry out HER in a strongly acidic solution and OER in a strongly alkaline solution, with bifunctional self-supported cobalt–nickel phosphide nanowire electrodes catalysing both reactions. Such asymmetric acid/alkaline water electrolysis could deliver a current density of 10 mAcm^−^^2^ with ca. 100% Faradaic efficiency at 1.567 V. Moreover, using an “irregular” BPM with unintentional crossover, the voltage needing to afford 10 mAcm^−^^2^ was reduced to 0.847 V due to the assistance of electrochemical neutralisation between acid and alkaline. J. Xu et al. [182] showed that BPM-based asymmetric water electrolysis could be accomplished in a circulated single-cell electrolyser delivering 10 mAcm^−^^2^ at 1.550 V and splitting water very stably for at least 25 h. Water electrolysis would be enabled by a solar panel operating at 0.908 V (@13 mAcm^−^^2^) using an “irregular” BPM. The results demonstrated that hydrogen production by BPM-based asymmetric water electrolysis is a promising alternative to conventional PEM and AEM water electrolysis.

A more integrated way to produce electrolytic hydrogen is through the chloralkali process, a major electrochemical technology based on the chloralkali industry. Hydrogen, chlorine, and sodium hydroxide are produced in the process. These compounds are used to manufacture a variety of intermediates in the chemical, pharmaceutical and crop protection industries. As the chloralkali industry is highly energy-intensive, electricity consumption represents a significant fraction (>90%) of the total production cost. The energy consumption depends on the electrolyser design, the type of membrane used, brine quality, and operating conditions. Thus, choosing the best membrane to install in a chloralkali electrolyser is a highly complex and relevant decision that should balance operating and fixed costs. Presently, the chloralkali membrane process is dominated by three membrane suppliers, and few studies are reported in the literature. This motivated A.C. Dias to carry out her PhD work [183] under the supervision of A. Mendes and to obtain valuable data for CUF—Químicos Industriais, S.A. (CUF-QI). This major Portuguese chloralkali company changed its name in 2018 to Bondalti. The work developed an experimental setup for determining the permselectivity of cation-exchange membranes and an electrochemical membrane reactor for studying the membrane cell process. It was demonstrated that significant energy savings could be achieved by choosing an adequate membrane and timely membrane replacement. The cost per ton of chlorine was calculated for three electrolysers, each using a set of a different type of membrane. It was concluded that the higher strength membrane had the lowest average total cost, with savings of around 1.5% compared to the second-best membrane. These data allowed a benchmarking methodology to be developed [183].

The same group used EIS to evaluate in situ the ohmic resistances of electrolytes and membrane in chloralkali membrane cells [184]. That allowed the effect of different operating parameters (current density, feed flow rate, brine and caustic concentrations, temperature) on the overall performance (power consumption) of a laboratory-scale chloralkali membrane cell to be assessed. It was found that cell temperature and brine concentration were the most critical variables on the cell voltage. The gas bubble evolution greatly affected the ohmic resistance, especially at the anode side, where the gas void fraction was about 10% [184].

**Figure 6:**
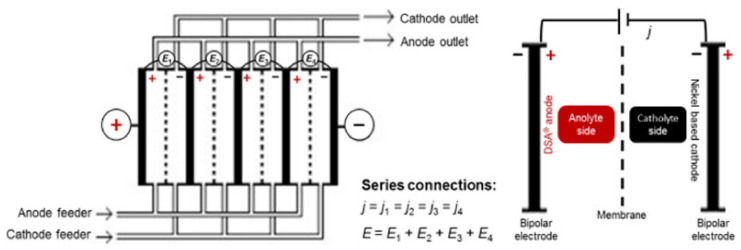
Scheme of a bipolar electrolyser where the stack consists of four cells connected in series: the potential between the terminals, *E*, is the sum of the individual cell potentials and the same current density, *j*, flows through the cells [185].

Franco et al. [185] carried out a systematic performance history analysis of a chloralkali membrane electrolyser under industrial operating conditions. The performance was analysed as a function of its service time for about 8 years, recording the cell potential versus current density. The ohmic overpotential contribution, associated with the ion-exchange membranes, showed an average relative error smaller than 3%. The activation overpotential, related to the electrodes’ performance, displayed an average relative error of 6%. This approach enabled assessing the performance of industrial chloralkali membrane electrolysers (Figure 6) for adequate maintenance scheduling, leading to significant operational and economic improvements in the chloralkali process [185].

### 3.4. Membranes for Gas Separation and Pervaporation

#### 3.4.1. Membranes for Gas Separation

Due to their close connection to global warming and unpredictable climate changes, anthropogenic greenhouse gas emissions are one of our era’s most critical environmental challenges. Specifically, carbon dioxide (CO_2_) is of particular concern as it currently accounts for the greatest portion of the warming associated with human activities. There are several means to curb the CO_2_ emissions to the atmosphere, including CO_2_ recovery from power plant flue streams (CO_2_/N_2_), CO_2_ separation from biohydrogen and fuel gas (syngas) in hydrogen production (CO_2_/H_2_), and CO_2_ removal from natural gas (natural gas sweetening) and biogas (CO_2_/CH_4_). However, the efficient separation of CO_2_ from light gases is the technical and economic key. Membrane technology can offer engineering and economic advantages compared to traditional separation methods, but the major drawback in current membrane operation concerns the limited selectivity of the materials. Hence, Portuguese researchers have intensively explored the development of advanced membrane materials for CO_2_ separation/capture.

Among the several membrane materials studied, a quite recent approach makes use of ionic liquids [186]. Remarkable CO_2_ solubility and tunability are essential features of this unique platform. The potential of ionic liquid materials to improve membrane-based CO_2_ separation processes has been demonstrated, either through the preparation of supported ionic liquid membranes (SILMs) [45,187,188,189,190,191,192,193,194,195,196,197,198], the blending of ionic liquids with poly(ionic liquid)s [199,200,201,202,203,204,205,206,207,208] or, more recently, through the development of mixed matrix membranes [209,210,211,212,213,214].

In a SILM, the selected ionic liquid is immobilised into the pores of an inert solid membrane supported by capillary forces. Inspired by the possibility of developing stimuli-responsive membranes with improved CO_2_ separation, Crespo’s group paid particular attention to magnetic ionic liquids containing metal-based anions with different magnetic susceptibilities in their structures [187]. The resulting SILMs exhibited CO_2_ permeabilities in the range of 147–259 Barrer, while CO_2_/N_2_ selectivity values between 21 and 41 were obtained. The same authors also focused on the permeability modulation of these SILMs when applying an external magnetic field (0–2 Tesla) [188]. The results showed that gas permeability increases as a function of the magnetic field depending on the metal anion, while CO_2_/N_2_ selectivity remained constant.

Aiming at full sustainability of CO_2_ separation processes, Tomé et al. [190] successfully prepared SILMs based on environmentally friendly cholinium carboxylate ionic liquids, which exhibited good CO_2_/CH_4_ selectivity (between 21 and 31) and CO_2_/N_2_ selectivity (from 39 to 50) at 20 °C under dry conditions. However, their CO_2_ permeability was significantly lower (2–18 Barrer) than that of other ionic liquid families. The major bottleneck of using cholinium carboxylates under dry conditions is their high viscosities, which lead to long absorption equilibrium times. With the intent of increasing the flexibility in tailoring the permeability and selectivity of SILMs for CO_2_ separation, Tomé et al. [189,191] explored the use of binary ionic liquid mixtures. The prepared SILMs revealed CO_2_/N_2_ separation performances on the respective Robeson upper bound, or even surpassed it, indicating their potential to separate CO_2_ in low-pressure post-combustion processes. The authors concluded that using ionic liquid mixtures combining the most selective anions with the least viscous anions is a promising strategy for designing advanced liquid phases for CO_2_ separation membranes. In this line, Gouveia et al. [193] proposed equimolar ionic liquid mixtures containing tricyanomethane and different amino acid-based to prepare SILMs for facilitated CO_2_ separation from flue gas streams. The best results were found at low trans-membrane pressure differentials (2.5 kPa), where the mixtures with _L_-alaninate and taurinate anions showed the highest CO_2_ permeability (765 Barrer) and CO_2_/N_2_ selectivity (69), respectively.

An innovative concept for removing CO_2_ from flue gas streams using SILMs was also proposed by Neves et al. [45]. This approach consisted of using an ionic liquid phase comprising a carbonic anhydrase (CAn) enzyme, which catalyses the conversion of CO_2_ to bicarbonate, enhancing the driving force for CO_2_ transport. Using this type of thermostable enzyme allowed us to obtain SILMs with high permeability towards CO_2_ (734 Barrer) at high temperature (up to 100 °C) and a reasonable transport selectivity towards CO_2_ against N_2_ (36) [195]. The use of CAn enzyme has recently been tested in supported liquid membranes using deep eutectic solvents (DES). For instance, a membrane containing CAn and DES composed of choline chloride and urea revealed high selectivity for CO_2_/CH_4_, setting above the respective Robeson upper bound [197]. In addition, a membrane prepared with CAn and a DES composed of choline chloride and levulinic acid showed a consistent behaviour up to 30 repeated cycles and stable operation at transmembrane pressure differences in the range of 0.5 to 1.25 bar [198]. These findings demonstrate the potential of DES-supported membranes for CO_2_ separation, particularly in the case of biogas.

**Figure 7:**
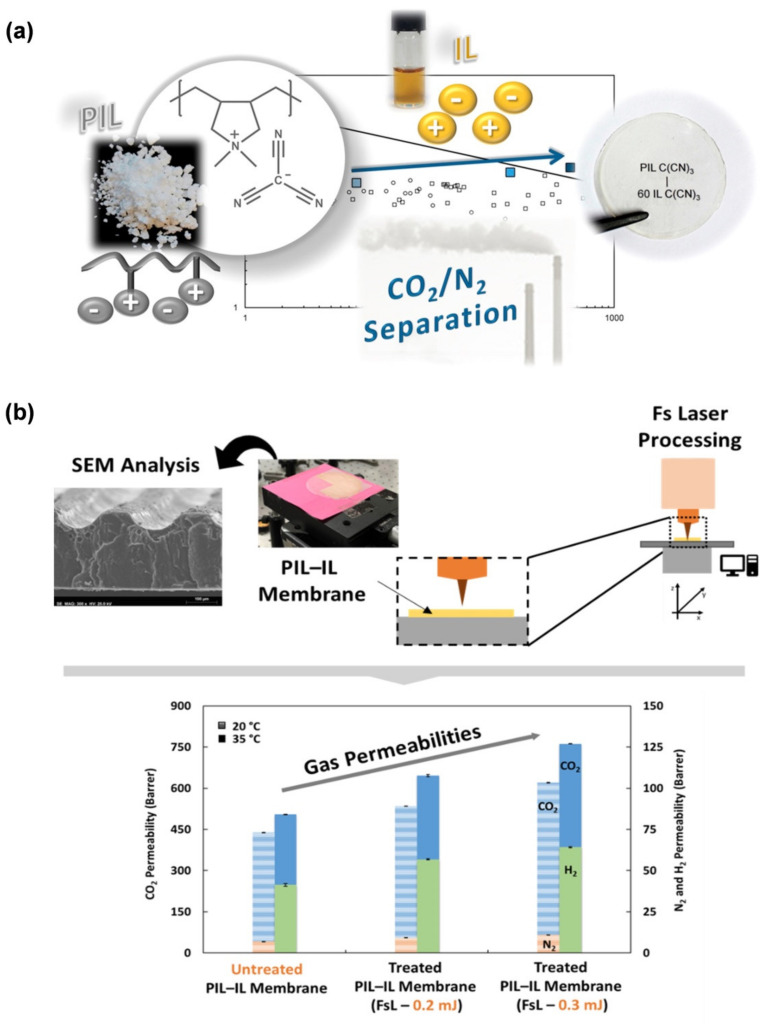
(**a**) CO_2_/N_2_ separation performance of PIL/IL membranes containing cyano-functionalised anions. Reprinted with permission from Tomé et al. [204] Copyright 2015, Elsevier. (**b**) Schematic illustration of the creation of microchannels on the surface of PIL/IL membranes by laser ablation using femtosecond laser radiation (top) and respective effect on permeability towards CO_2_, N_2_, and H_2_ (bottom). Reprinted with permission from Gouveia et al. [208]. Copyright 2022, Elsevier.

Another strategy for designing membranes for CO_2_ separation is the fabrication of poly (ionic liquid) (PIL)–ionic liquid (IL) membranes. This strategy combines the intrinsic ionic liquid properties with the macromolecular structure of PILs, taking advantage of their ability to strongly interact through electrostatic interactions. Tomé et al. [199] investigated PIL/IL membranes based on PILs having different polycation functionalities (i.e., imidazolium, pyridinium, pyrrolidinium, ammonium, and cholinium) and selected the pyrrolidinium-based PILs to conduct different studies since their synthetic route is less complicated than those of the other PIL polycations. The results showed that the CO_2_ permeability and selectivity of PIL–IL membranes bearing pyrrolidinium polymer matrices could be adjusted by varying the IL content [200], using random copolymers with anion mixtures [201] or PIL and IL components with different anions [202], and even by changing the polymer molecular weight [203]. The best performances were obtained for PIL/IL membranes containing cyano-functionalised anions, particularly the one with 60 wt.% of [C_2_mim][C(CN)_3_] IL, which surpassed the Robeson 2008 upper bound for CO_2_/N_2_ separation (Figure 7a), with CO_2_ permeability of 440 Barrer and CO_2_/N_2_ selectivity of 64 [204].

Pyrrolidinium-based PIL/IL membranes have also been successfully tested for CO_2_/H_2_ separation in hydrogen purification [205,206,207]. Despite the competition effect between gases in mixed gas experiments, only small differences were obtained between mixed and ideal CO_2_/H_2_ selectivities. The pyrrolidinium-based PIL/IL membranes showed mixed CO_2_/H_2_ separation performances above the upper bound even at the highest temperature (80 °C) and feed pressure (4 bar) tested. Once more, the PIL C(CN)_3_–60 [C_2_mim][C(CN)_3_] membrane showed the best mixed gas performance, with CO_2_ permeability of 325 Barrer and CO_2_/H_2_ selectivity of 11.4 [207]. Furthermore, Gouveia et al. [208] have recently explored the creation of microchannels (around 55–60 μm deep) on the surface of PIL/IL membranes by laser ablation using femtosecond laser radiation (Figure 7b). Considerable improvements in CO_2_, N_2,_ and H_2_ permeabilities were achieved for irradiated PIL/IL membranes, while their CO_2_/N_2_ and CO_2_/H_2_ selectivities were almost unchanged.

Mixed matrix membranes combining metal-organic frameworks (MOFs) [209,210,211,212] or graphene [213,214] have also been investigated. As an example, Nabais et al. [209] incorporated different MOFs (MIL-53(Al), Cu_3_(BTC)_2_, and ZIF-8) into a pyrrolidinium-based PIL/IL (60/40 wt.%). These multi-component (PIL/IL/MOF) membranes surpassed the upper bound limit for CO_2_/H_2_ separation, with the one loaded with 30 wt.% of ZIF-8 showing CO_2_ permeability of 97, while that of MIL-53(Al) presenting CO_2_/H_2_ selectivity of 13. Nevertheless, using the PIL/IL/MOF-5 system for CO_2_/CH_4_ separation revealed performance limitations, possibly attributed to poor chemical/physical compatibility between the PIL/IL matrix and MOF-5 particles [210].

Furthermore, inorganic porous materials have been explored for different kinds of gas mixtures [215,216,217,218,219]. In particular, carbon molecular sieve (CMS) membranes are strong candidates for energy-efficient gas separation applications, due to their known higher separation performances, stability to corrosive environments and temperatures (up to ca. 200 °C). Potential applications include carbon dioxide removal from natural gas/biogas streams, hydrogen recovery from streams with light hydrocarbons, light olefins/paraffins separation, xenon recovery and air separation, among others [220]. Mendes’ group pioneered in Portugal the development of CMS membrane technology through the work of Lagorsse et al. [62,63], who studied the transport mechanism and the ageing effect of the CMS membranes modules produced by Carbon Membranes Ltd., an Israeli company in this field. Teixeira et al. [65] produced CMS membranes from low-cost phenolic resin, with a record C_3_H_6_ permeability of 420 Barrer and a C_3_H_6_/C_3_H_8_ selectivity of 18.1, making these membranes a promising alternative for olefins purification. Later on, Rodrigues et al. [221] prepared renewable and low-cost cellophane-based CMS membranes with the highest reported O_2_/N_2_ selectivity (>800), CO_2_/CH_4_ and CO_2_/N_2_ (>2600), and H_2_/CH_4_ and H_2_/N_2_ greater (>25,000). Tailor-made CMS membranes with a well-developed microporous structure were also prepared by Rodrigues et al. [222] from an optimised, low-cost and renewable ionic liquid-regenerated cellulose. The CMS membranes surpassed the Robeson upper bound for O_2_/N_2_, He/N_2_ and H_2_/N_2_ separations [222]. Very recently, Araújo et al. [67] fabricated stable cellulose-based CMS membranes, also from an ionic liquid regenerated cellulose precursor, with extremely high selectivities to H_2_/CH_4_ and CO_2_/CH_4_ (>206,000 and 14,600, respectively), due to the precise pore size. The produced CMS membranes upon a propylene post-treatment for 10 days present an ageing resistant property for oxygen chemisorption for more than 1 year without impairing the CMS membrane performance [67]. These results demonstrate the industrial attractiveness of this type of membrane for the separation of gases with high performance and stability.

#### 3.4.2. Membranes for Pervaporation

Pervaporation is a membrane separation process with unique and adequate features to remove volatile compounds present in liquid mixtures at trace levels [223]. Pervaporation has been used by Portuguese researchers for the dehydration of organic solvents (such as ethanol [224,225,226,227], isopropanol [50] or n-butanol [228]), aroma recovery [49,87,229,230,231,232,233,234], and process intensification of ethyl lactate [235] or butyl acrylate [213,236].

**Figure 8:**
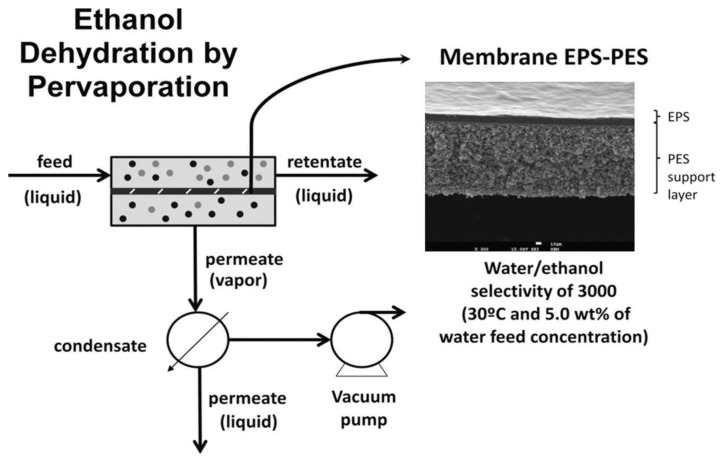
Scheme of ethanol dehydration by pervaporation (left) and the composite membrane of the crosslinked EPS supported in polyethersulfone (EPS-PES) (right). Reprinted with permission from Meireles et al. [224]. Copyright 2013, Elsevier.

Solvent dehydration processes have a high economic and environmental significance in the pharmaceutical, fine chemistry, and chemical industries. Hydrophilic pervaporation has gained progressive recognition among the available techniques, particularly for ethanol dehydration [226]. In this process, hydrophilic membranes are preferred due to their high selectivity for water, but they also may present poor mechanical stability. As an alternative, Meireles et al. [224] proposed a new microbial exopolysaccharide (EPS) membrane obtained from a low-cost and abundant carbon source. The composite membranes of the crosslinked EPS supported in polyethersulfone (EPS-PES) showed high potential for ethanol dehydration, with a water/ethanol selectivity of 3000 at 5.0 wt.% water concentration in the feed (Figure 8). The effect of biopolymer purification was also studied. The EPS-PES membrane prepared with polysaccharides purified by dia-ultrafiltration was revealed to be the best choice, with a water/ethanol selectivity of 142 for 10 wt.% water in ethanol [225]. Meireles et al. [227] also developed hybrid polysaccharide (FucoPol) membranes by incorporating a SiO_2_ network. Although the hybrid FucoPol membranes showed high water selectivity values (similar to PERVAP 4101^®^ commercial membrane), their long-term operation was compromised by an irreversible swelling in the presence of water.

Organophilic pervaporation is a particularly suitable process for aroma recovery since it does not require the use of any additional extracting agent and operates under mild conditions. Just to cite some examples, Brazinha et al. [230] proposed an integrated process for recovery and capture of aroma compounds from dilute natural matrices combining the advantages of sweeping gas pervaporation with gas–liquid absorption in membrane contactors. Several potential food/cosmetic compatible additives were tested, and high partitions of the aroma compounds to poly(ethylene glycol) (PEG) 300 were observed [230]. Kulkarni et al. [231] demonstrated that organophilic pervaporation enabled an efficient concentration of limonene with fewer contaminants and lower water content when compared to traditional vacuum distillation. The direct recovery of natural vanillin from post-fermentation media was also studied and optimised by Brazinha et al. [232], assuring the quantitative recovery of vanillin free of other media constituents in a single pervaporation step.

Another important contribution concerns the use of pervaporation extracting equilibrated aromas. In particular, Catarino et al. [234] used asymmetric composite membranes of polyoctylmethylsiloxane/polyetherimide (POMS/PEI) for aroma extraction from beer by pervaporation. This work allowed Super Bock Group, S.A. (formerly Unicer Bebidas S.A.) to apply this technology to develop a new industrial process for producing non-alcoholic beer with a corrected natural flavour profile [88]. This new process removes the aromas by pervaporation, and the beer without aroma is fed to an industrial distillation unit in a rotating cone column for dealcoholisation. Finally, the non-alcohol beer is again mixed with the aromas previously collected by the pervaporation unit. This process allowed the company to produce a non-alcoholic beer with an ethanol content of less than 0.5% by volume without harming the initial flavour of this product [88]. Furthermore, Catarino et al. [87] proposed an identical process for dealcoholising wine. The authors tested reverse osmosis and nanofiltration membranes to remove alcohol content from wine and pervaporation membranes to recover the frames. The use of this combined process has proven to be effective in removing alcohol from wine without impairing its taste [87].

In addition, pervaporation processes are strongly recommended for heat-sensitive products and azeotropic mixtures, as in the case of the butyl acrylate system. Constantino et al. [236] presented pervaporation data for multicomponent mixtures in the absence of reaction for the compounds involved in the esterification of acrylic acid with n-butanol at different temperatures. This experimental data is very useful for designing and optimising pervaporation-based hybrid processes for butyl acrylate synthesis [213].

## 4. Future Perspectives

Research is universal and, naturally, membrane research in Portugal is aligned with global concerns and expectations for sustainable social development. The United Nations Development Goals translate in a clear way the principles that are guiding us towards inclusive and sustainable development.

Considering these principles, it is natural that membrane research in Portugal is progressively focusing on the use of natural resources and renewable energy. Several examples can be given, such as:

- The development of membranes from natural, renewable resources, namely plant and bacterial cellulose and other natural (bio)polymers, which can be modified and functionalised for a diversity of applications;
- The recovery of chemical elements that are becoming rare and are required for many applications associated with a sustainable lifestyle: (bio)refining of wastewaters aiming the recovery of essential elements such as phosphorus; sea mining, seeking the recovery of a large diversity of elements, including lithium and rare metals;
- The use of renewable sources of energy, namely the integration of solar and membrane technology, but also the exploitation of salinity gradients for the harvesting of energy, supported on membrane-assisted processes.

The development of new membranes, seeking for new or improved functionalities, assuring their sustainable production, extending their service time and life cycle is one major trend on membrane research in Portugal:

- Membranes with new functionalities, including research for membranes with controlled pore size (i.e., isoporous) and pore size distribution, with extremely high hydrophilicity or extremely high hydrophobicity namely for membrane contactors applications, highly resistant to organic solvents, namely using natural polymers as starting raw materials, and responsive to external stimuli (photo and magnetic stimuli are among approaches under study) aiming different applications, notably in the biomedical area;
- Development and fabrication of membranes using less toxic, sustainable solvents, such as supercritical fluids, room temperature ionic liquids and deep eutectic solvents, among others;
- Recycling/reusing membranes after use, namely by modifying their properties extending their service time.

Additionally, research towards intensified processes, although assuring their sustainability, are critical issues under development:

- Intelligent use of energy, avoiding processes that involve phase transition and applying energy where required: at the interface where mass and energy transfer takes place. For this purpose, membrane research aiming the design of membranes with a designed topography that promotes turbulence near their surface, assuring improved mass and heat transfer conditions, are currently under development;
- Module design, as well as their components, using computational fluid dynamics is also a hot subject, aiming higher throughput by assuring better mass and heat transfer conditions, and minimising fouling with a minimum expenditure of energy. Spacer design and new (revisited) fouling control strategies are under consideration, such as the use of rotating and vibrating membranes.
- Efficient monitoring of membrane processes, seeking on-line, real-time monitoring, making possible the establishment of advanced automation and better process control strategies. This approach may require the use of different sources of information/monitoring signals, some of them rather complex that, using data-driven modelling and machine learning approaches, may lead to advanced control with an impact on process efficiency;
- Process integration in close interaction with corporate partners aiming the implementation and validation of membrane processes in all relevant domains with impact in social development: safe food and water production, valorisation and management of waste (regarded as by-products), environmental protection, energy production and storage, production of goods and health protection.

We would like conclude by emphasising the commitment of the membrane community in Portugal in education and awareness programmes at all levels, which translates our concern to develop knowledge in membrane science and engineering, transposing it to welfare and social development.

## Abbreviations

2D	two-dimensional
AEM	anion-exchange membrane
ASI	Advanced Study Institute
BioPEM	biopolymer electrolyte membrane
BNC	bacterial nanocellulose
BPM	bipolar membrane
CA	cellulose acetate
CAn	carbonic anhydrase
CFD	computational fluid dynamics
CMS	carbon molecular sieve
CNC	cellulose nanocrystal
CNF	cellulose nanofibril
DBFC	direct borohydride fuel cell
DCMD	direct contact membrane distillation
DD	Donnan dialysis
DES	deep eutectic solvent
DF	dia-ultrafiltration
DLFC	direct liquid fuel cell
DMFC	direct methanol fuel cell
EEM	excitation-emission matrix
EIS	electrochemical impedance spectroscopy
EPS	exopolysaccharide
FCT NOVA	Nova School of Science and Technology
FEUP	Faculdade de Engenharia da Universidade do Porto
HER	hydrogen evolution reaction
IEMB	ion-exchange membrane bioreactor
IL	ionic liquid
IST	Instituto Superior Técnico
LIB	lithium-ion battery
LNEC	Laboratório Nacional de Engenharia Civil
MBO	membrane blood oxygenator
MEA	membrane-electrode assembly
MF	microfiltration
MFC	microbial fuel cell
MOF	metal-organic framework
NAqRFB	non-aqueous redox flow battery
NF	nanofiltration
NOM	natural organic matter
OER	oxygen evolution reaction
OMW	olive mill wastewater
OTC	oxytetracycline
PAA	poly(acrylic) acid
PAC	powdered activated carbon
PCA	principal components analysis
PCL	polycaprolactone
PDMS	polydimethylsiloxane
PEG	poly(ethylene glycol)
PEI	polyetherimide
PEM	proton exchange membrane
PES	polyethersulfone
PIL	poly (ionic liquid)
POMS/PEI	polyoctylmethylsiloxane/polyetherimide
PU	polyurethane
PVA	poly(vinyl alcohol)
PVDF-TrFE	poly(vinylidene fluoride-co-trifluoroethylene)
RED	reverse electrodialysis
RO	reverse osmosis
SILM	supported ionic liquid membrane
SGO	sulphonated graphene oxide
sPEEK	sulfonated poly (ether ether ketone)
SS	silica spheres
UF	ultrafiltration
